# Electrical stimulation of the bed nucleus of the stria terminalis reduces anxiety in a rat model

**DOI:** 10.1038/tp.2017.2

**Published:** 2017-02-14

**Authors:** K Luyck, T Tambuyzer, M Deprez, J Rangarajan, B Nuttin, L Luyten

**Affiliations:** 1 Division of Experimental Neurosurgery and Neuroanatomy, KU Leuven, Leuven, Belgium; 2 Division of Animal and Human Health Engineering, KU Leuven, Leuven, Belgium; 3 Medical Imaging Research Center, KU Leuven, Leuven, Belgium; 4 Department of Neurosurgery, UZ Leuven, Leuven, Belgium; 5 Centre for Psychology of Learning and Experimental Psychopathology, KU Leuven, Leuven, Belgium

## Abstract

We recently showed that deep brain stimulation (DBS) in the bed nucleus of the stria terminalis (BST) reduces obsessions, compulsions and associated anxiety in patients suffering from severe, treatment-refractory obsessive-compulsive disorder. Here, we investigated the anxiolytic effects of electrical BST stimulation in a rat model of conditioned anxiety, unrelated to obsessions or compulsions. Two sets of stimulation parameters were evaluated. Using fixed settings at 100 Hz, 40 μs and 300 μA (Set A), we observed elevated freezing and startle levels, whereas stimulation at 130 Hz, 220 μs and individually tailored amplitudes (Set B) appeared to reduce freezing. In a follow-up experiment, we evaluated the anxiolytic potential of Set B more extensively, by adding a lesion group and an additional day of stimulation. We found that electrical stimulation significantly reduced freezing, but not to the same extent as lesions. Neither lesions nor stimulation of the BST affected motor behavior or unconditioned anxiety in an open-field test. In summary, electrical stimulation of the BST was successful in reducing contextual anxiety in a rat model, without eliciting unwanted motor effects. Our findings underline the therapeutic potential of DBS in the BST for disorders that are hallmarked by pathological anxiety. Further research will be necessary to assess the translatability of these findings to the clinic.

## Introduction

Anxiety disorders are among the most prevalent psychiatric disorders, and cause substantial disability and suffering.^1, 2^ Over the years, multiple research groups have attempted to unravel the complex circuits and mechanisms that underlie fear and anxiety. Fear is commonly described as a phasic response in the presence of an imminent, specific threat; whereas anxiety is considered a sustained response in the possibility of future threat, triggered by diffuse stimuli.^3, 4^ In the lab, fear can be acquired by pairing an explicit cue (for example, a tone) with an aversive event (for example, a shock); while administering unpredictable, unsignaled shocks results in sustained anxiety.^5^

Multiple structures have been shown to be involved in the network that underlies anxiety,^6, 7^ including the amygdala,^8, 9^ prefrontal cortex,^10, 11, 12^ hippocampus,^13, 14^ nucleus accumbens^15, 16^ and, more recently, the medial forebrain bundle.^17, 18^ Together with the amygdala, the bed nucleus of the stria terminalis (BST) is one of the most extensively studied structures in this regard.^4, 19, 20, 21, 22, 23, 24^ Although both structures are highly similar in terms of inputs, outputs and neurochemical make-up,^25^ the amygdala appears to mediate phasic fear responses, whereas the BST is involved in the expression of sustained anxiety.^4^ This intriguing behavioral distinction was first described by Hitchcock and Davis, who showed that lesions of the central amygdala (CeA) reduced the phasic fear response, while BST lesions did not.^8, 9^ Reversely, BST, but not CeA, lesions reduced sustained anxiety responses in the light-enhanced startle paradigm.^21^ Other studies confirmed this distinction, using different conditioning protocols.^22, 26, 27^

The involvement of the BST in anxiety also carries translational importance. Imaging studies revealed a hyperactive BST region when human subjects were anticipating adverse events (for example, electrical shock or phobia-related stimuli).^28, 29, 30^ In addition, deep brain stimulation (DBS) in the BST region has emerged as a last-resort treatment option for patients suffering from severe, treatment-resistant obsessive-compulsive disorder (OCD).^31, 32^ A long-term follow-up study confirmed that BST stimulation is safe and effective in two-thirds of these patients in decreasing obsessions, compulsions and associated anxiety and depressive symptoms.^24, 33^

In most OCD patients, anxiety-evoking thoughts are inherent to their obsessions and compulsions.^34^ Moreover, the therapeutic effects of DBS in the BST region in these patients may be primarily driven by its anxiolytic effect.^24^ In the current study, we evaluated whether electrical BST stimulation reduces acquired anxiety in a rat model that is not confounded by the presence of obsessions and compulsions. In particular, we used a context conditioning procedure, which represents some key aspects of pathological anxiety (that is, lingering, unpredictable threat)^5, 35, 36^ and therefore holds clinical relevance.^37^

## Materials and methods

First, we investigated whether our conditioning procedure evokes context-specific anxiety that requires associative learning, rather than mere sensitization due to shock exposure. Second, we explored different sets of electrical stimulation parameters and evaluated their effects on anxiety measurements. Finally, we assessed stimulation effects in a follow-up experiment, where we included motor behavior and unconditioned anxiety measured in an open field.

### Subjects

Male Wistar rats (±250 g, 8 weeks old) were used in experiment (Exp) 1 (*n*=16), in Exp2A (*n*=32), Exp2B (*n*=32) and Exp3 (*n*=48). All the animals were housed in pairs with food and water available *ad libitum*. For Exp 2 and 3, a plastic cage divider was used to prevent damage to the surgical wound by cage mates, while still allowing for social interaction. The animals were maintained on a 14/10 h light–dark cycle (lights on at 0700 h), with a room temperature of ±19 °C. This project was in accordance with the Belgian and European laws, guidelines and policies for animal experimentation, housing and care (Belgian Royal Decree of 29 May 2013 and European Directive 2010/63/EU on the protection of animals used for scientific purposes of 20 October 2010).

### Equipment

In all experiments, the animals were conditioned in a small animal cage (inner dimensions: 9.4 cm height, 8.2 cm width and 16.5 cm length) with a grid floor, through which foot shocks were delivered. The grid floor consisted of six 5-mm-diameter stainless-steel bars spaced 10 mm apart (MED Associates, Fairfax, VT, USA). Note that the startle cage was adapted using a customized 3D-printed, pyramidal top lid, to allow for insertion of stimulation cables in Exp2 and 3. The cage was fixed on a response platform and located inside a ventilated sound-attenuating box (MED Associates). A red light bulb (3.8 W) was continuously on. The freezing behavior of the animals was recorded by a video camera (DCR-SR55E Super NightShot Plus; Sony, Tokyo, Japan) positioned in front of the test cage. In addition, the startle reaction of the rats generated a pressure on the response platform and analog signals were amplified, digitized, and processed by software (Startle Reflex, version 5.95; MED Associates). The presentation and sequencing of the acoustic stimuli and foot shocks were controlled by the same software. One of two loudspeakers, both located 7 cm behind the rat holder, was used to deliver a continuous white background noise (55 dB); the other speaker delivered the startle stimuli (white noise, 100 dB, 50 ms). The startle response was defined as the first peak accelerometer voltage that occurred during the first 100 ms after onset of the startle probe and was measured on an arbitrary scale ranging from 0 to 2047. The startle platform and loudspeakers were calibrated before each experiment. The cage was cleaned with 70% ethanol between rats.

### Context conditioning protocol

We used a context conditioning protocol with dual behavioral measurements (freezing and startle response) that has been described previously.^27^ All the experimental steps were strictly timed using ExpTimer software.^38^

#### Habituation

On the first day, the rats were placed in the startle box for a total of 20 min. During the first 5 min (acclimation phase), only background noise (55 dB) was presented. Afterwards, 30 acoustic startle stimuli (100 dB, 50 ms) were administered with a fixed intertrial interval of 30 s. This habituation phase was added to stabilize startle responses, before any experimental manipulations took place. The data obtained from this test phase were not included in our analysis.

#### Pre-test

On day two, the rats underwent a pre-test identical to the habituation session. On the basis of their pre-test startle values, the rats were matched into equivalent groups for all the experiments. In this phase, we collected our baseline measurements of anxiety, that is, freezing and startle.^39, 40^ Percentage freezing during the 5-min acclimation phase was scored manually by an observer (KL), blinded to the group division. The startle measurements were collected automatically from the Startle Reflex software.

#### Training

After a 5 min acclimation phase, all the rats received 10 electrical foot shocks (0.8 mA, 250 ms) with a variable intertrial interval of 60–180 s. At this stage, the rats were conditioned to the context. The total duration of the training session was 30 min.

#### Post-test

On day 4, the animals were tested using the 20 min protocol identical to that of habituation and pre-test. During post-test, the animals are expected to express anxiety to the context in which they previously received electrical shocks, as measured by increased startle and freezing during acclimation.

### Experiment 1: Contextual anxiety in conditioned rats

Context conditioning was conducted according to the standard protocol described above, with the exception that training was either carried out in the same (SAME) or a different (DIFF) test cage than that used for testing. The ‘DIFF' training context consisted of a cage (21 cm height, 25 cm width, 30 cm length), located in a sound-attenuating box (MED Associates). The cage had a standard grid floor with 19 bars and a black triangular ceiling.^41^ The box was dimly lit with a white light of 50 lux and the cage was cleaned with a scented cleaning product between rats.

### Experiment 2: Electrical stimulation in the BST with fixed or individual parameters

Custom-made monopolar electrodes (127 μm diameter Pt/Ir rods, AM Systems, Sequim, WA, USA) were implanted under general anesthesia (ketamine hydrochloride (22.5 mg kg^−1^, Anesketin, Eurovet nv/sa, Heusden-Zolder, Belgium) and 0.15 mg kg^−1^ medetomine HCL (Kela, Sint-Niklaas, Belgium)). The rats were placed in a stereotaxic frame and a craniotomy was performed. Two burr holes were drilled to allow for bilateral electrode insertion in the BST (anterior–posterior: 0.0 mm; mediolateral: ±1.2 mm, 5.9 mm subdurally). Four stainless-steel screws (Fine Science Tools, Heidelberg, Germany) were inserted in the skull through smaller burr holes. Dental cement (Tetric EvoFlow, Ivoclar Vivadent, Mississauga, ON, Canada) was used to cover the electrodes and the fixation screws before suturing the wound. Throughout the surgery, the body temperature of the rats was monitored through an anal probe and adjusted by a feedback-controlled heating pad (Harvard Apparatus, Holliston, MA, USA). Postoperative pain treatment (Metacam, 1 mg kg^−1^, Boehringer Ingelheim Vetmedica, Ingelheim/Rhein, Germany) was injected subcutaneously after surgery. The animals were allowed to recover for 6–7 days before the start of behavioral experiments.

The standard conditioning procedure was followed. On each day of the behavioral protocol, all the animals were connected to the stimulation set-up (DS8000 and DLS100, World Precision Instruments, Stevenage, UK and 363-SL/6, Plastics One, Roanoke, VA, USA). Actual stimulation (biphasic, bilateral stimulation) only took place during post-test, for animals in the STIM group. To evaluate and allow for attenuation of potential side effects, stimulation was initiated in the home cage 1 h before the post-test. Two sets of stimulation parameters were evaluated. In Exp2A, we used a frequency of 100 Hz, a pulse width of 40 μs and a fixed amplitude of 300 μA (Set A), which was chosen within the range of commonly used settings throughout rodent literature.^42, 43, 44^ In Exp2B, frequency and pulse width were fixed at 130 Hz and 220 μs, respectively (Set B). The amplitude was determined for each animal individually, by increasing in 50 μA steps to the level where side effects were observed (for example, excessive shaking, jaw or paw spasms, freezing, extensive urination and defecation). The amplitude was then lowered gradually, until the animal resumed its normal behavior, and this amplitude was used throughout the post-test (highest tolerable amplitude). This approach was chosen to correspond to clinically used parameters that are generally successful in achieving therapeutic effects in OCD patients receiving DBS in the BST region.^24^ In fact, minor side effects such as flushing and facial muscle twitching are observed in several patients.^32, 45, 46^ Slightly decreasing the stimulation amplitude induces symptom relief without side effects, indicating that determining the highest tolerable amplitude is a valuable strategy.

### Experiment 3: Comparison of anxiolytic effects of electrical stimulation and electrolytic lesions in the BST

Twenty-four animals were implanted with monopolar electrodes in the BST, as described for Exp2. Another group of 24 animals received stainless-steel cannulas (23-gauge guide cannula C317G/5mm and dummy stylet C317DC/5mm, Plastics One) on the dura, directed towards the BST (anterior–posterior: 0.0 mm, mediolateral: ±3.4 mm, 20° angle to the sagittal plane). In addition, a mock pedestal was fixated on the skull to allow for connection of the rats to the stimulation device, therefore correcting for cable interference. The animals were allowed to recover for 6–7 days before the start of behavioral experiments.

In this experiment, the standard 4-day conditioning protocol was extended with a reminder training (two shocks, 90 s intertrial interval, to diminish extinction) 2 min after the post-test and with a second post-test on day 5, which allows us to examine whether the effects of electrical stimulation are consistent over two testing days. Rats implanted with electrodes were divided between CTRL (*n*=7) and STIM (*n*=17) groups, rats implanted with cannulas were assigned to CTRL (*n*=9) and LES (*n*=15) groups. Using this approach, the CTRL group contained rats that underwent either the pedestal or cannula (with mock pedestal) implantation, and additionally, the blinded experimenter could not infer group division based on headstage type during behavioral analyses.

Three hours after the end of the training session, all the rats were briefly anesthetized with isoflurane (5% (induction) and 2% (maintenance) in 1.5–2.0 liter min^−1^ oxygen). LES rats received bilateral BST lesions, as described previously.^27^ Rats implanted with cannulas belonging to the CTRL group underwent the same process, but received sham stimulation at 0 mA. Rats implanted with electrodes (STIM and CTRL group) were also anesthetized, to correct for potential interference of anesthesia. The lesion procedure was performed by MD, while KL remained blinded to the group division throughout the experiment.

On post-test 1 and 2, the STIM rats received electrical stimulation 1 h before and during both post-tests with the same stimulation parameters as those used in Exp2B (130 Hz, 220 μs and individual amplitudes). Note that stimulation was ceased before the additional reminder shocks were administered after post-test 1.

On day 6, the rats underwent a 10 min open-field test (80 × 80 × 80 cm, black floor and walls, ±250 lux) to evaluate effects of BST manipulations on both motor behavior (‘Total distance' and ‘%Movement') and innate anxiety (‘Time in center'). The center of the open field was defined as 25% of the total surface. Movement and location were detected by a video algorithm developed by Tambuyzer *et al.*^47, 48^ The rat was considered to be moving when the change in its centroid position exceeded 5 mm within 1 s. All the rats were connected to a stimulation cable, while only STIM animals were actually stimulated 1 h before and during open-field testing.

### Histology

**Figure 1:**
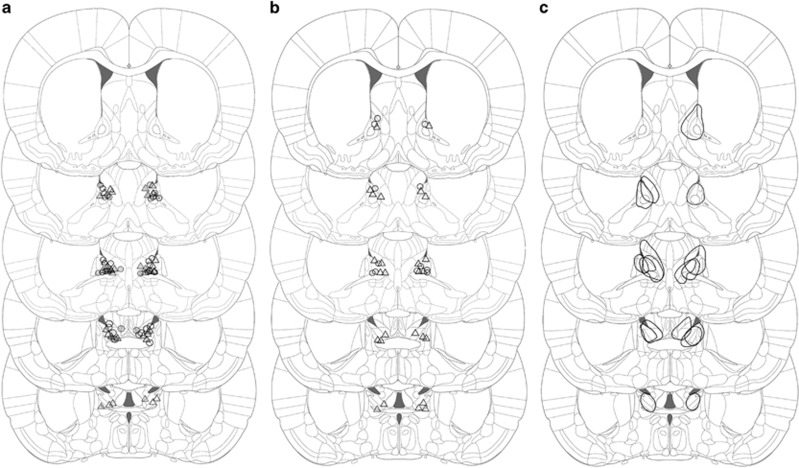
Reconstruction of electrode tips and lesions of all rats included in the analyses. (**a**) shows electrode tips of Exp2A (gray) and Exp2B (black and white). (**b**) shows electrode tips of Exp3 and (**c**) shows the maximal diameter of each lesion in Exp3. Circles represent CTRL rats, triangles correspond to STIM animals. Coronal slices shown from top to bottom are +0.48 mm, +0.24 mm, 0.00 mm, −0.24 mm and −0.48 mm, with respect to bregma. Figure adapted from Paxinos and Watson, 2005.^67^

Approximately 1 week after testing, all the rats of Exp2 and Exp3 were given a lethal injection of pentobarbital (2 ml intraperitoneal, Nembutal, CEVA Santé Animale, Brussels, Belgium). The animals were perfused with a solution of 10% sucrose (D(+)-Saccharose, VWR International bvba, Leuven, Belgium), and subsequently with a 4% formaldehyde solution (37% dissolved in water, stabilized with 5–15% methanol, Acros organics, Geel, Belgium, 10 × diluted in deionized water). The brains were dissected and stored in 4% formaldehyde, processed and embedded in paraffin. Five micrometer thick coronal slices were collected with the microtome (Leica Biosystems, Nussloch, Germany) and stained with Cresyl-Violet (0.5% cresyl violet acetate in dH2O, Merck, Darmstadt, Germany). The microscopical analysis revealed the exact location of electrode tips and lesions, which were transferred to a two-dimensional Paxinos slice. The electrode position was accepted within a 500 μm radius surrounding the target coordinates (anterior–posterior: 0.00 mm, mediolateral: ±1.2 mm, 5.9 mm subdurally). Lesion animals were included when clear BST damage (including necrosis and edema) was visible on the bregma slice (Figure 1).

### Statistical analyses

All data are represented as means±s.e.m. (GraphPad Prism, version 4.03; GraphPad Software, San Diego, CA, USA). Statistical analyses were performed with Statistica (Statistica 9; StatSoft, Tulsa, OK, USA). Assumptions were met for all the tests and the sample sizes were determined based on previously conducted stimulation experiments in our group. Significance levels were set at *P*<0.05.

#### Exp1 and Exp2

The pre-test startle measurements were analyzed using an unpaired *t*-test, to verify successful matching between groups. The post-test freezing and startle measurements were corrected for baseline values on the pre-test and are therefore shown as difference scores (post-pre). These difference scores of ‘% Freezing' and ‘Startle' were compared between groups (SAME vs DIFF in Exp1; STIM vs CTRL in Exp2) by means of an unpaired *t*-test.

#### Exp3

A one-way analysis of variance (ANOVA) was used to compare pre-test startle measurements between the CTRL, STIM and LES groups. To evaluate context conditioning in Exp3, we performed a two-way repeated-measures ANOVA for ‘% Freezing' and ‘Startle'. The CTRL, STIM and LES groups (factor ‘Group') were compared over two time points, which were normalized to pre-test: post1-pre and post2-pre (factor ‘Session'). Tukey's *post hoc* analysis was performed to specify group differences. Finally, open-field behavior was analyzed by a one-way ANOVA to examine group differences between CTRL, STIM and LES rats for ‘% Time in center', ‘Total distance' and ‘% Movement'.

## Results

### Exp1: Contextual anxiety in conditioned rats

**Figure 2:**
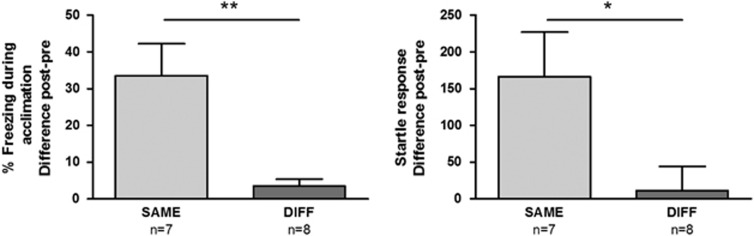
Percentage freezing during acclimation (left panel) and startle response (right panel) in rats that were trained and tested in either the same (SAME) or a different context (DIFF). Difference scores of post-test minus baseline (pre-test) are shown (mean±s.e.m.). *P<0.05, **P<0.01.

One rat of the SAME group was excluded from all analyses, due to equipment malfunction. Pre-test startle measurements were not significantly different between SAME (*n*=7) and DIFF (*n*=8) groups (*t*_(13)_=−1.14; *P*=0.27), indicating that matching was effective. In addition, pre-test freezing was low in all the animals (1.9%±2.3%). At post-test, rats in the SAME group froze significantly more than the DIFF group (*t*_(13)_=−3.61; *P*<0.01) and showed higher startle values (*t*_(13)_=−2.32; p=0.04). Moreover, freezing and startle potentiation were negligible in DIFF rats (Figure 2). Taken together, this indicates that the post-test anxiety in our standard conditioning procedure (SAME group) is context-bound and not merely an effect of sensitization or general arousal, but the result of an associative learning process.

### Exp2A: Electrical stimulation in the BST with fixed parameters

Four animals lost their headstage during the training session, and 10 animals (CTRL: *n*=5; STIM: *n*=5) were excluded based on incorrect electrode placement. Eighteen animals were included in the final analysis (CTRL: *n*=8; STIM: *n*=10) (Figure 1a).

**Figure 3:**
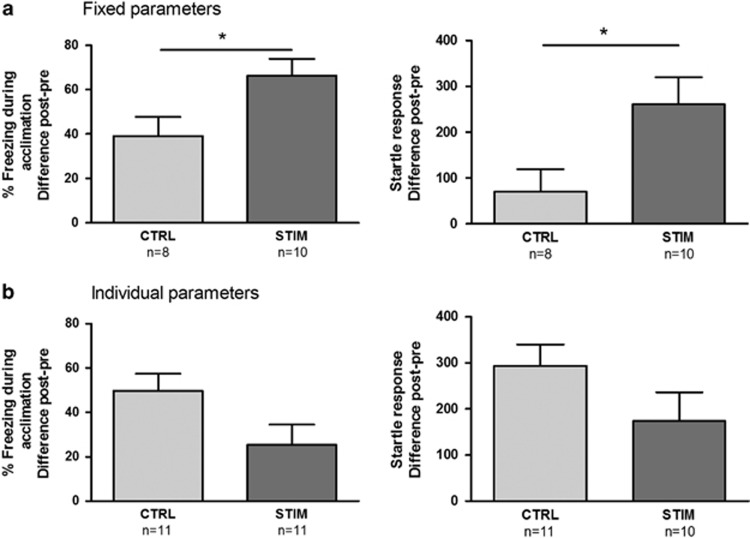
Effects of electrical bed nucleus of the stria terminalis (BST) stimulation on contextual anxiety using fixed (**a**) and individual (**b**) stimulation parameters in Exp2A and 2B, respectively. Anxiety was indexed with percentage freezing during acclimation (left panels) and startle response (right panels). Difference scores of post-test minus pre-test are shown (means±s.e.m.). *P<0.05.

Pre-test startle was comparable in CTRL and STIM groups (*t*_(16)_=−0.59; *P*=0.56) and freezing values were low (5.6%±7.7%). Both freezing and startle levels were substantially increased during post-test (Figure 3a). Animals in the STIM group froze significantly more than CTRL animals (*t*_(16)_=2.33; *P*=0.03) and displayed higher startle values (*t*_(16)_=2.41; *P*=0.03), implying an anxiogenic effect of electrical stimulation.

### Exp2B: Electrical stimulation in the BST with individual parameters

Six animals lost their headstage during behavioral testing and four were excluded due to incorrect electrode placement (CTRL: *n*=1; STIM: *n*=3). For freezing, we included all the remaining animals (CTRL: *n*=11; STIM: *n*=11) (Figure 1a). For startle measurements, one additional animal was excluded due to equipment malfunction (CTRL: *n*=11; STIM: *n*=10).

Pre-test startle values were comparable between CTRL and STIM animals (*t*_(19)_=0.24; *P*=0.81) and freezing values were low (2.5%±3.2%). Post-test freezing and startle measurements were elevated compared with pre-test, and did not differ between groups (*t*_(20)_=−2.02; *P*=0.057 and *t*_(19)_=−1.54; *P*=0.14, respectively; Figure 3b). Although no significance was reached, we decided to investigate the anxiolytic trend seen in the freezing data in a more extensive follow-up experiment.

### Exp3: Comparison of anxiolytic effects of electrical stimulation and electrolytic lesions in the BST

One rat died during lesion induction, probably due to isoflurane intolerance, and four animals lost their headstage during context conditioning (CTRL: *n*=2) or open-field testing (STIM: *n*=2). On the basis of histological analyses, we further excluded one CTRL, two STIM and five LES animals. In addition, one animal of the LES group was excluded because it had not sufficiently recovered 1 day after lesion induction (porphyrin discharge around eyes and nose, puffy appearance, immobile—these are presumably aspecific side effects of anesthesia or electrode insertion). In summary, 34 subjects were included for context conditioning (CTRL: *n*=12; STIM: *n*=13; LES: *n*=9) and 32 for open-field analysis (CTRL: *n*=12; STIM: *n*=11; LES: *n*=9) (Figure 1b, c).

**Figure 4:**
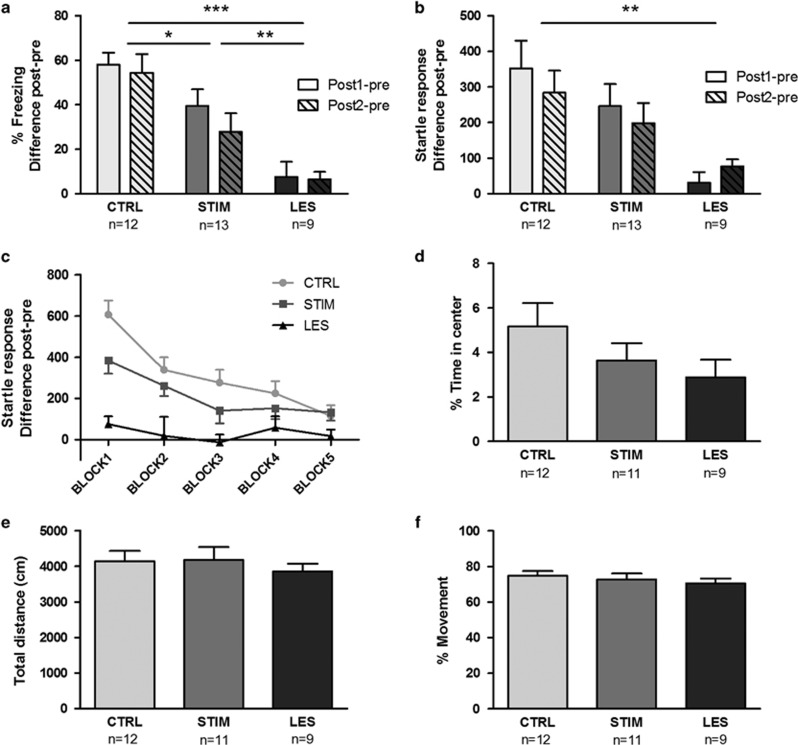
Effects of bed nucleus of the stria terminalis (BST) stimulation and BST lesions on context conditioning (**a**–**c**), unconditioned anxiety (**d**) and motor behavior (**e** and **f**) in Exp3. Percentages freezing during acclimation and startle responses are shown in **a** and **b**, respectively, using difference scores of post-tests minus pre-test. Significant differences between groups are indicated. In **c**, the time course of the anxiolytic effects of BST stimulation is illustrated. Startle responses were divided into five blocks of six startle probes each, during the 15 min test period after acclimation. CTRL and STIM groups represent pooled data of Exp2B and Exp3. The percentage of time the animals spent in the center of the open field is represented in **d**. The total distance traveled and percentage movement during open-field testing is shown in **e** and **f**, respectively. Data are shown as means±s.e.m. *P<0.05, **P<0.01, ***P<0.001.

The pre-test startle values were comparable between groups (F_(2,31)_=0.23; *P*=0.79) and the freezing levels were low (3.3%±6.8%). The analysis of freezing during acclimation revealed a main effect of ‘Group' (F_(2,31)_=16.94; *P*<0.0001). Tukey's *post hoc* analysis showed that lesioned rats froze less than the STIM and CTRL animals (*P*<0.01 and *P*=0.0001, respectively). In addition, the STIM animals froze less than the CTRL animals (*P*=0.02; Figure 4a). These results indicate that stimulation in the BST is indeed anxiolytic, but not to the extent of BST lesions. In concordance with the freezing data, startle analysis revealed a main effect of ‘Group' (F_(2,31)_=5.77; *P*<0.01). Tukey's *post hoc* revealed that startle levels were significantly lower in lesioned animals, compared with CTRL (*P*<0.01). The STIM values were numerically lower than CTRL and higher than LES values, but did not differ significantly from either (Figure 4b).

Next, we examined the anxiolytic effects observed in Exp2B and Exp3 in more detail, by plotting the startle response of Post-test 1 in five blocks of six startle probes each (Figure 4c). Significant group differences were only reached in LES vs CTRL (F_(2,52)_=6.20; *P*<0.01). However, we observed that rats receiving electrical stimulation, on average, displayed lower startle values in the first four blocks compared with CTRL animals, with the highest nominal difference being present in block1. An exploratory one-way ANOVA confirmed a significant group effect (F_(2,52)_=10.70; *P*=0.0001) during this block. The CTRL rats displayed higher startle values in block1 compared with STIM and LES rats (*P*=0.04 and *P*<0.001, respectively). In addition, LES rats had lower startles compared with STIM (*P*=0.03). These data suggest that electrical stimulation primarily affects the initial expression of anxiety when the anxious memory is retrieved, rather than enhancing extinction. In the open-field test, no group differences were found for ‘% Time in center' (F_(2,29)_=1.63; *P*=0.21; Figure 4d), ‘Total distance' (F_(2,29)_=0.29; *P*=0.75; Figure 4e) or ‘% Movement' (F_(2,29)_=0.54; *P*=0.58; Figure 4f), indicating that BST manipulations did not affect motor behavior or innate, unconditioned anxiety.

## Discussion

In this study, we demonstrated that electrical stimulation in the BST reduces contextual freezing in a rat model of anxiety.

In Exp1, we demonstrated that the elevated freezing and startle responses indeed represent contextual anxiety, and not just mere sensitization because of shock exposure on the preceding day. In the following experiments, we used the context conditioning model to investigate the effect of BST stimulation on acquired anxiety, which has relevance for various anxiety disorders.^37, 49^

To our knowledge, few studies are available on BST stimulation in models of (contextual) anxiety. Van Dijk *et al.*^43^ found no effect of BST stimulation on (un)conditioned anxiety. As the authors indicate themselves, their conditioning protocol was unsuccessful in evoking sustained anxiety, which may account for their negative results in terms of the effects of BST stimulation. In another study, Baas *et al.*^50^ recently examined the effect of electrical stimulation on conditioned anxiety in OCD patients receiving DBS. No effects were found on contextual anxiety, as indexed by fear-potentiated startle. However, some neuroanatomical and paradigm-related concerns that confound interpretation of their results should be taken into account (see ref. 51 for discussion), leaving the effects of human BST stimulation on contextual anxiety open for investigation. Finally, Rodriguez-Romaguera *et al.*^42^ showed that stimulation of the dorsomedial ventral striatum facilitates extinction of cued fear, whereas stimulation of the ventrolateral part of the ventral striatum impairs extinction. The authors did not evaluate the effects of stimulation on sustained anxiety in a context conditioning procedure with unsignaled shocks. Moreover, their stimulation target was located more anteriorly than ours and comprised the nucleus accumbens rather than the BST.

In the current study, we explored two sets of stimulation parameters in the BST and evaluated their effect on the expression of contextual anxiety. In Exp2A, we found that stimulation at fixed settings (Set A: 100 Hz, 40 μs, 300 μA) increased both startle and freezing responses compared with CTRL animals. Note that the higher startle responses in the stimulated group should be interpreted with caution because of relatively low values in the CTRL group. Nevertheless, the effects of stimulation on freezing are clear and suggest increased anxiety, although we cannot rule out that these particular stimulation settings perturbed the animals' general well-being (for example, more freezing due to headache).

In Exp2B, no significant effects on either freezing or startle were found using individual settings (Set B: 130 Hz, 220 μs and individual amplitude). However, we observed a trend toward anxiolytic effects, with lower freezing values in the STIM group (*P*=0.057), in line with the nominally, but not significantly, lower startle responses. Note that several animals had to be excluded due to technical difficulties, thereby decreasing the group size and potentially accounting for the absence of a significant effect. Interestingly, the anxiolytic trend in Exp2B is opposite to the effects obtained in Exp2A. Multiple studies have underlined the importance of careful parameter optimization to achieve symptom alleviation.^52, 53, 54^

In Exp3, we evaluated the individualized parameters more extensively by adding a BST lesion (LES) group as a positive control,^22, 27^ and including a second post-test to evaluate the consistency and replicability of stimulation effects. We found that electrical stimulation significantly reduced freezing, and reduced startle responses in the first startle block. As the extinction process gradually gains importance over time, we postulate that electrical stimulation is more likely to affect the expression of anxiety than to enhance extinction. Electrical stimulation significantly decreased freezing, but electrolytic lesions had superior effects on both freezing and startle values.^55, 56^ However, DBS holds several advantages over permanent lesions, as it allows for adjustable, individual parameter settings to achieve optimal therapeutic effects with minimal side effects. From a clinical perspective, a reversible and adaptable neurosurgical procedure is preferable, but further research will be pivotal to optimize DBS effects and evaluate the potential of this treatment option for patients suffering from pathological anxiety. A few considerations have to be taken into account when interpreting the results obtained in Exp3. As DBS is a curative instead of a preventive technique, we chose to stimulate during expression rather than during acquisition of anxiety. STIM animals received stimulation only 1 h before and during the post-test sessions, whereas the lesion in LES animals was made 3 h after training, to allow sufficient time for the animals to recover from the lesion procedure.^27^ Although unlikely, we cannot rule out that the lesion may also interfere with the consolidation phase, thereby partly accounting for its superior effects compared to the stimulation group. In addition, electrolytic lesions may also destroy white matter tracts, thereby leading to distal effects beyond the target structure. In this study, we used electrolytic lesions due to their clinical relevance (for example, capsulotomy in OCD patients) and for comparability with our previous studies.^27^ Moreover, others have already demonstrated the anxiolytic effects of fiber-sparing BST lesions.^9, 57^ As a final remark, data from the second post-test should be interpreted with caution. This additional post-test allowed us to evaluate stimulation parameters on multiple test days and increase statistical power without including more animals. However, both post-tests are not identical. Post-test 2 is influenced by extinction and reminder shocks on post-test 1 and could therefore recruit different brain structures. In addition, lesions were already present when the reminder shocks were given, therefore complicating direct comparison with CTRL and STIM groups on post-test 2. Nonetheless, freezing and startle responses appear constant on both post-tests (Figures 4a and b, respectively) underlining the usefulness of the second post-test.

To ensure that the anxiolytic effects obtained in Exp3 were not confounded by motor effects, we evaluated locomotion in an open field. We found that BST manipulations had no effect on the total distance traveled and the percentage movement during this 10 min test. Overall, there seems to be a consensus that BST inactivation does not interfere with motor behavior,^58, 59, 60, 61, 62^ but also see ref. 43. In addition, it is unlikely that pure motor effects could explain our results, as both freezing and startle measurements appear highly consistent within each experiment. Although increased motor behavior may explain reduced freezing, it would not account for decreases in startle measurements, or the other way around. This underlines the relevance of using a protocol with combined measurements of anxiety.^40^ Finally, we showed that neither BST lesions nor stimulation had an effect on unconditioned anxiety, measured by the time spent in the center of an open-field test, indicating that the effects of our BST manipulations may be specific to acquired anxiety.

Finally, we must take into consideration that the BST is a highly complex structure, which entails subdivisions that may account for opposing effects on anxiety.^19, 20^ In addition, electrical stimulation of the BST probably not only affects the BST itself, but also surrounding structures within a millimeter range, including fiber tracts passing through the stimulated area.^63^ In this regard, it is noteworthy that DBS in the NAc region also alleviates symptoms (including anxiety) in OCD patients,^16^ although BST stimulation is believed to have superior effects.^24, 64^ Pre-clinical studies have shown that fiber-sparing BST lesions reduce anxiety, whereas similar lesions in the NAc cannot replicate the effects obtained by electrical stimulation in this region.^42^ This suggests that the BST in itself is responsible for anxiolytic effects, whereas the NAc might serve as an integration center through which the BST interacts with (mostly) cortical areas. Recently, it has been suggested that anxiety-related brain structures, such as the BST, should be added to our neuroanatomical models of the circuits underlying OCD.^24, 65, 66^

Here, we demonstrated that electrical stimulation in the BST reduces anxiety in a rat model without typical OCD-related obsessions and compulsions. Taken together, we argue that the reduced anxiety levels obtained in OCD patients through BST stimulation are a primary effect of DBS, rather than a ‘passive' consequence of reduced obsessions and compulsions. In concordance, Denys *et al.*^16^ described a sequential order for symptoms alleviation, starting with reduction of depressive and anxiety symptoms (within minutes), whereas obsessions and compulsions took days or weeks to improve. Given the existing clinical experience with BST stimulation in OCD patients and our current findings, we suggest that DBS in the BST may provide a new treatment option for patients suffering from severe anxiety disorders, such as generalized anxiety or posttraumatic stress disorder.

In conclusion, we demonstrated that electrical stimulation of the BST reduces acquired anxiety in a rat model. Further research will be necessary to evaluate the potential of DBS in the BST as a last-resort treatment option for anxiety patients. In addition, our findings lay the foundation for a more fundamental investigation of the mechanisms of DBS in the BST.
